# Prognostic Potential of the Body Composition Indices in Predicting Positive Changes in Resting Blood Pressure after High-Intensity Interval Training in Adolescents

**DOI:** 10.3390/ijerph192214658

**Published:** 2022-11-08

**Authors:** Jarosław Domaradzki, Dawid Koźlenia, Marek Popowczak

**Affiliations:** 1 Unit of Biostructure, Faculty of Physical Education and Sport, Wroclaw University of Health and Sport Sciences, Al. I. J. Paderewskiego 35, 51-612 Wroclaw, Poland; 2 Unit of Team Sports Games, Faculty of Physical Education and Sport, Wroclaw University of Health and Sport Sciences, Al. I. J. Paderewskiego 35, 51-612 Wroclaw, Poland

**Keywords:** body composition indices, resting blood pressure, high-intensity interval training, ROC analysis, area under the curve, cardiovascular parameters improvement

## Abstract

This study aimed to investigate the prognostic potential of body composition indices in predicting the improvement in resting blood pressure after 10 weeks of high-intensity interval training (HIIT) implemented in physical education lessons. The participants were 141 adolescents aged 16 years. Independent variables were body mass index (BMI), fat mass index (FMI), muscle mass index (SMI), and mass to fat ratio (MFR); dependent variables were systolic and diastolic blood pressure (SBP, DBP) and its indices: pulse pressure (PP), mid-blood pressure (MBP), and mean arterial pressure (MAP). The receiver operating curve (ROC) method was employed. SMI and MFR are body composition indices with prognostic potential to predict positive changes in SBP in males (SMI: AUC = 0.82; *p* < 0.001, MFR = 0.70; *p* = 0.039) and MFR in females (AUC = 0.72; *p* = 0.035). The respective cut-off point values used to classify participants as a beneficiary of HIIT intervention concerning SBP were SMI = 7.84 and MFR = 2.43 in males, and for SMI = 10.12 and MFR = 1.94 in females. Body composition indices based on skeletal muscle (SMI, MFR) were more likely to predict positive changes in SBP after HIIT intervention in adolescents. PP, MBP, and MAP did not reflect the detecting power of SMI and MFR. However, these thresholds’ utility is limited to adolescents of 16 years of age.

## 1. Introduction

Appropriate systolic blood pressure (SBP) is 120 mmHg, and diastolic blood pressure is 80 mmHg in adults, with a slight difference in childhood and adolescence. A person with SBP over 140 mmHg and/or DBP over 90 mmHg is diagnosed with hypertension. Blood pressure exceeding the normal range is a serious medical problem that significantly increases the risk of many diseases, e.g., metabolic syndrome, cardiovascular diseases, and heart diseases [1]. Global statistics show 10.4 million deaths due to hypertension [2]. The prevalence of elevated blood pressure among youth is associated with numerous diseases before adulthood and premature mortality [3,4]. More and more young people suffer from abnormalities in blood pressure [5,6]. Evidence suggests the need to look for factors underlying elevated blood pressure and effective methods of counteracting the abnormalities to prevent hypertension.

The worldwide prevalence of obesity in the early stage of life is already well-documented [7,8,9]. Many reports suggest a direct and strong link between body fat and the state of blood pressure [10,11,12]. The interrelationship between fatness and elevated blood pressure enhances additive adverse health effects [13]. 

Fatness can be considered as a distribution in different body locations (measured among others with waist-to-hip ratio (WHR)) or quantity of fat mass in the whole body (body fat mass (BFM) in kilograms or percentages (BFP)) in different parts of the body (trunk, higher, or lower extremities). Even though the measurements are interpreted differently, they are related to health. Excessive adipose tissue (adiposity) is a reason for adverse endocrine responses provoking cardiovascular diseases [14]. Furthermore, excessive visceral adiposity resulted in elevated blood pressure. It was the main reason for metabolic disorders [15,16]. In contrast, muscle mass was independently and inversely associated with elevated blood pressure and cardiovascular disturbances [17,18]. A biological mechanism is based on the protective effect of high muscle quality on cardiovascular health [19]. 

Evidence that excessive fatty tissue and inadequate muscle mass–fat mass proportions are the risk factors of blood pressure disturbances obliges us to examine different body weight indices as predictors of elevated blood pressure or hypertension. Body mass index (BMI) and waist-to-hip ratio (WHR) is the most often used. However, they have limitations concerning specific groups of people (physically active, sports athletes, military persons, etc.) [20,21]. Therefore, other indices that can predict blood pressure changes are examined. 

An emerging alternative for assessing fat tissue in relation to height or muscle mass is the fat mass index (FMI), skeletal muscle index (SMI), and muscle-to-fat ratio (MFR) [22]. All of that indices have recently gained significance, owing to several factors, including the similarity of its calculation method to that of BMI [23]. Their prognostic potential in detecting some phenomena, e.g., the risk of injuries, the risk of osteoporosis, or positive changes in physical efficiency, was positively verified [24,25,26,27,28,29]. 

Among the instruments for fighting with excessive fat tissue and elevated blood pressure, physical activity comes to the fore. The great value of physical activity, which leads to normal body mass and normotensive blood pressure, has been proved many times [30,31,32]. The problem is that young people, particularly adolescents, are generally reluctant to undertake PA in their leisure time. Therefore, physical education (PE) lessons seem to be relevant settings to implement physical activity for adolescents [33]. Bond et al. [34] implemented short, time-efficient high-intensity interval training (HIIT) in PE and received positive results related to decreasing the risk of cardiovascular diseases. Similar effects were observed in body fat mass (lowering BFM) and blood pressure (reduction in SBP and not so clearly in DBP) [35,36,37,38]. HIIT reduces body fat, improves physical efficiency, and increases muscle mass [39]. Positive effects were observed among obese adolescents, where HIIT improved body composition and cardiovascular parameters [40,41]. Additionally, Delgado-Floody et al. [42] confirmed significant improvement in anthropometric and cardiovascular parameters after 28 weeks of HIIT intervention.

However, current Polish studies on associations between body weight and its components with resting blood pressure are limited mainly to the assessment of the simple relationship between the weight-to-height ratios, waist and hip circumferences, and body fat with resting blood pressure but do not include the analysis of various fat mass indices (FMI, SMI, MFR). Moreover, to the best knowledge of the authors, to date, there is no assessment of the predicting usefulness of the body composition indices in detecting positive changes in resting blood pressure after high-intensity interval training programs implemented in regular PE lessons. Our study focused on evaluating the various body composition indices based on anthropometric measurements (waist-to-hip ratio (WHR), body mass index (BMI), fat mass index (FMI), skeletal mass index (SMI), and muscle-to-fat ratio (MFR)) as predictors of the cardiovascular state after 10 weeks of regular short-term intensive physical effort in an average adolescent population. Furthermore, the utility of various blood pressure indices (pulse pressure (PP), mid-blood pressure (MBP), and mean arterial pressure (MAP)) were tested apart from systolic (SBP) and diastolic (DBP) blood pressure parameters. Therefore, the study’s primary purpose was to investigate the prognostic potential of these body composition indices in predicting the improvement in resting blood pressure after ten weeks of HIIT implemented in PE lessons in adolescents. Specifically, we aimed to: (1) assess the utility of each index in prediction (based on AUC), and (2) identify the associated thresholds in adolescents.

## 2. Materials and Methods

Results in this study were obtained from data received in the project “Physical activity and nutritional education in preventing civilization diseases—theoretical aspects and practical implications for the secondary school physical education program”. The full description of the project and methodology was published elsewhere [38,43,44]. Here, only brief information was presented.

### 2.1. Participants and Study Design

The sample size was calculated before starting the project, based on primary planned analysis—MANOVA—and it was identified that 179 participants should be included. Examinations were conducted in school where there were 187 students in the 1st year of school learning. In total, 46 of them did not finish intervention or were excluded due to various reasons (e.g., involving in organized physical activity, medical contradiction, etc.). Thus, the final sample size of the participants comprised 141 adolescents. They were separated randomly into an experimental group (EG) and a control group (CG). 

**Figure 1:**
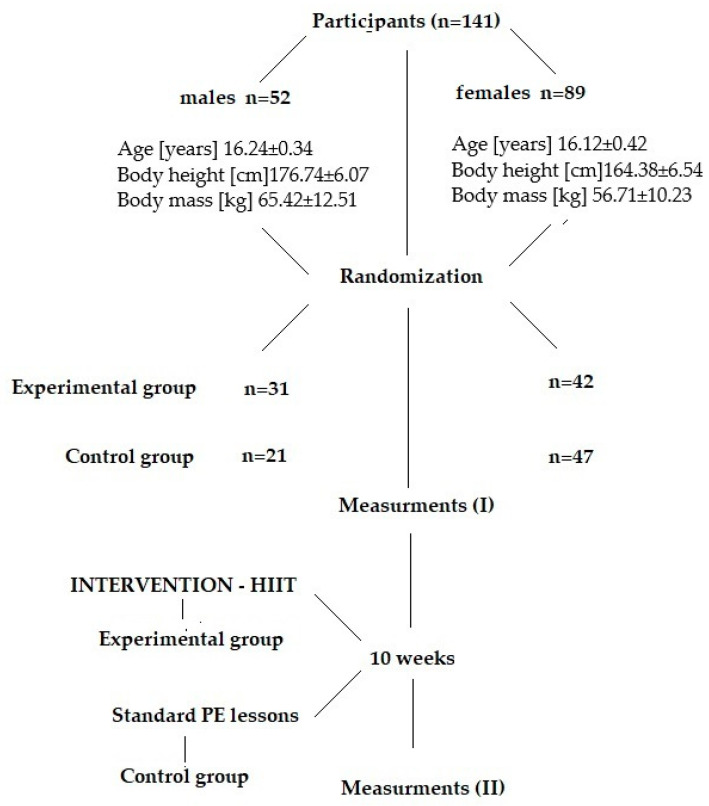
Descriptive characteristics of the participants and study design.

Figure 1 presents descriptive characteristics considering the participants’ mean age, body height, and mass and study design. All subjects were from the same comprehensive secondary school in Wroclaw, Poland. 

### 2.2. Intervention

High-intensity interval training (HIIT) based on the Tabata protocol (TAP) was conducted for ten weeks. Participants qualified for the experimental group performed the TAP during one weekly PE lesson. A standard PE lesson (45 min) started with a standardized 10 min warm-up. The main activity part was 14 min of TAP, which comprised three sessions, each lasting four minutes (eight cycles of two exercises: push-ups and high knees in the first session; dynamic lunges and spider crawl in the second session; plank to push-ups and side squeeze in third one) [45]. Maximum-intensity exercise lasting 20 s of as many repetitions as possible and then a 10 s active rest and a 1 min break between each session were performed. 

Exercise intensity during the TAP with adolescents’ maximum heart rate was determined with the formula HRmax = 208 − 0.7 × age (16 years) established [46]. High-intensity exercise ranged from 75% to 80% of the maximum heart rate was 145–157 bpm. A polar H1 heart rate monitor was used to monitor students’ heart rates during the first PE lesson with TAP (Polar Electro, Kempele, Finland).

### 2.3. Procedures

Measurements were taken twice: baseline (preintervention variables) and after the 10-week intervention (postintervention variables). Examinations were conducted one day from 8:00 a.m. to 1:00 p.m. in the same order: anthropometric and body composition measurements and resting blood pressure after 10 min of rest. 

### 2.4. Anthropometric and Body Composition Measurements

Body height measurement was taken with an accuracy of 0.1 cm using an anthropometer (GPM Anthropological Instruments). Body weight and body fat mass were measured with the InBody230 body composition analyzer (InBody Co. Ltd., Cerritos, CA, USA). Waist and hip circumferences were measured with the standard anthropometric procedure. The above data were used to calculate indices with the presented formulas:

WHR = w/h, where: w—waist circumference [cm], h—hip circumference [cm],

BMI = bw/bh^2^, where: bw—body weight [kg], bh—body height [m],

FMI = bfm/ bh^2^, where: fm—body fat mass [kg], bh—body height [m],

SMI = sm/bh^2^, where: sm—body skeletal muscle mass [kg], bh—body height [m],

MFR = sm/fm, where: sm—body skeletal muscle mass [kg], fm—body fat mass [kg]

### 2.5. Resting Blood Pressure

This paper used results of resting blood pressure before and after a 10-week intervention. Automatic Blood Pressure Monitor—Omron BP710 tool was used to measure systolic (SBP (bpm)) and diastolic (DBP (bpm)) blood pressure. Before measurement, participants were asked to sit quietly for 10 min. Readings of resting SBP and DBP were taken three times in 10 min intervals. The mean value of the three measurements was recorded in the database.

Based on SBP and DBP blood pressure, various indices were calculated too. The following formulas were used [47]:

PP (pulse pressure—the difference between SBP and DBP) = SBP − DBP,

MBP (mid-blood pressure—an average of SBP and DBP) = (SBP + DBP)/2,

MAP (mean arterial pressure—weighted means) = 2 × DBP + SBP/3

MBP and MAP are weighted means with constant weights, with MBP giving equal weight to SBP and DBP, while MAP shows SBP as a weight of one-third and DBP as a weight of two-thirds. Both MBP and MAP indices were verified to perform better than SBP and DBP in predicting various disorders [48,49].

### 2.6. Data Analysis

Statistical characteristics of the analyzed variables in EG and CG and detailed comparisons between groups were presented elsewhere [39,43,44]. In this paper, descriptive statistics (mean and 95% CI) of baseline body composition indices and differences between pre-and post-intervention blood pressure indices (Δ) were presented. T-Student tests were conducted between EG and CG (for males’ and females’ groups). Delta variables were converted into a binomial scale: 1—positive changes (negative values if the post-intervention value was lower than the pre-intervention value) and 0—lack of changes (and vice versa). 

The numbers of positive changes, as well as the lack of changes, were presented as percentages. Pearson’s chi-square test (χ^2^) was conducted to assess the association between positive changes and lack of changes with experiment (EG–CG comparison) and sex (males–females comparison). Odds ratios (OR) for assessing the likelihood of the positive changes for groups were calculated too. 

Next, the receiver operating curve (ROC) method was employed to assess the utility of the body composition indices in predicting positive changes in blood pressure indices. The accuracy of body composition measures to discriminate positive changes in groups of participants was assessed using the area under curve (AUC) parameter with confidence interval (95% CI). AUC statistic measures the model’s goodness of fit and validity based on sensitivity and specificity. Differences between AUC values between each pair of body composition indices were tested using DeLong tests [50].

For statistically significant models, the cut-off points were calculated. It was done using the Youden index, calculated from the formula:

*J* = *maximum* {*sensitivity* + *specificity* − 1}; *overall cut-points c*; −∞ < *c* < ∞.

The Youden index allows, based on the ROC curve sensitivity and specificity values, the optimal cut-off point to be determined [25,26].

A *p*-value < 0.05 was considered statistically significant. The calculations were carried out using Statistica 13.0 (StatSoft Poland 2018, Cracow, Poland).

## 3. Results

Descriptive statistics of raw measurements and calculated indices (body composition and blood pressure) are presented in Table 1. Detailed comparisons between EG and CG groups using t-Student tests showed no differences in baseline body composition indices and significant changes in blood pressure after intervention in blood pressure parameters in EG groups (Table 1).

Out of 141 students, 87 (61.70%) decreased SBP, and 78 (55.32%) decreased DBP during the period of lasting the program (independently of the group—experimental or control). In consequence, positive changes in blood pressure indices were: PP—74 (52.48%), MPB—87 (61.70%), and MAP—84 (59.57%).

Considering subgroups (experimental and control), students from experimental groups noted improvements in blood pressure parameters much more often than peers not included in the program. It was supposed to be linked with the intervention effect. It regards SBP for both sex (83.87% EG males vs. 38.10% CG males, χ^2^ = 11.59, *p* < 001; 83.33% EG females vs. 38.30% CG females, χ^2^ = 18.67, *p* < 0.001), DBP for females (69.05% EG females vs. 38.30 CG females, χ^2^ = 11.59, *p* < 0.001; males—differences not significant), PP for males (74.19% EG males vs. 42.86% CG males, χ^2^ = 5.19, *p* = 0.023), MBP for both sexes (74.19% EG males vs. 47.62% CG males, χ^2^ = 3.81, *p* = 050; 78.57% EG females vs. 44.68% CG females, χ^2^ = 10.68, *p* = 0.001) and MAP for females (73.81% EG females vs. 42.55 CG females, χ^2^ = 8.86, *p* = 0.003; males—differences not significant). Calculated odds ratio (OR) showed that males and females who participated in the intervention program were several times more likely to improve blood pressure parameters than peers from control groups. The highest value was observed for SBP (males: OR = 8.45 (2.30–31.03 95% CI); females: OR = 8.05 (2.95–21.94 95% CI)).

The next step of the analysis was to assess the diagnostic potential of the body composition indices in predicting the improvement of blood pressure. AUC with attending statistics was calculated. ROC curves for experimental and control males and females were drawn for the most accurate body composition and blood pressure indices or parameters. 

The diagnostic accuracy of the cut-off points for predicting positive changes in cardiovascular parameters by indices using skeletal muscle mass in the equation was higher than what would be expected by chance (AUC > 0.5) for all indices except DBP (Table 2). Body mass index and fat mass index were not good predictors, and their utility in detecting positive changes in the cardiovascular system was limited. In the EG males’ group, the best predictive potential received SMI (AUC = 0.82, *p* < 0.001) and MFR (AUC = 0.70, *p* = 0.039), while in EG females, it was only MFR (AUC = 0.72, *p* = 0.035). Results for CG groups were not significant.

Comparing the differences between AUC values, SMI showed significant differences compared to BMI (*p* = 0.041) and FMI (*p* = 0.014) for SBP and FMI (0.044) for MBP in EG males, and to FMI in EG females (*p* = 0.050) (Table 3). Results for CG groups were not significant.

**Figure 2:**
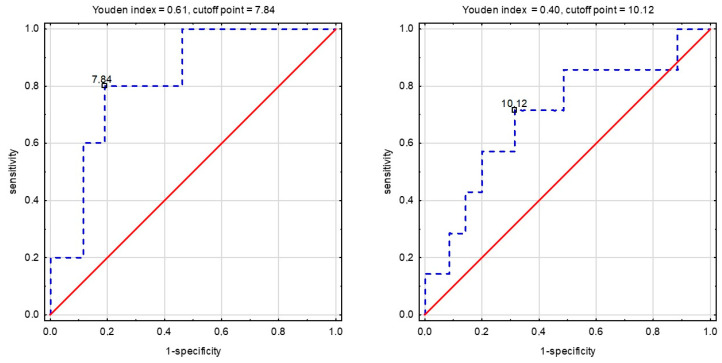
The receiver operating characteristic curve for skeletal muscle index predicts positive changes in SBP in experimental group (EG) males and experimental group (EG) females.

Cut-off point values were calculated only for statistically significant ROC curves. Figure 2 illustrates the ROC curve for SMI, which presented the highest and statistically significant AUC. Cut-off point values characterized by the highest sensitivity and specificity in SBP for SMI and MFR were: 7.84 (males) and 10.12 (females), and 2.43 (males) and 1.94 (females), respectively.

## 4. Discussion

The purpose of the paper was twofold: first, examine the utility of various body composition indices in predicting positive changes in the cardiovascular system (for different blood pressure indices) in adolescents; second, identify cut-off point values for body composition indices best-performing prediction. Regarding the first objective, indices based on skeletal muscle mass (SMI and MFR in males, MFR in females), but not BMI and FMI, effectively distinguished between positive changes and lack of changes concerning SBP. Regarding the second objective, thresholds for values of skeletal muscle mass concerning body height (SMI) and body fat (MFR) reflected the chance of positive changes in the cardiovascular system (particularly systolic blood pressure). Interventions based on aerobic and anaerobic exercise are widely used in programs related to human health [51,52,53,54]. Particularly, high-intensity interval training is an effective method for improving health parameters considering body composition and hemodynamics parameters of physical fitness (martin smith). The mentioned above factors are connected. Therefore, the HIIT effect is broad, and its implementation in physical education significantly influences adolescents [12]. However, adults and the elderly may benefit from HIIT or any similar program [55]. 

A significant reduction of cardiovascular risk after the HIIT program caused by reducing blood pressure was observed in overweight participants [56]. Additionally, it was shown that after HIIT, besides improving cardiovascular parameters, was observed reduction in fat mass [57]. Some authors suggest that HIIT or other short intensive efforts may be more effective in decreasing cardiovascular risk than prolonged, moderate-intensity activities [58]. Kouuba et al. [59] confirmed this result and demonstrated more benefits after HIIT in body composition, cardiorespiratory fitness, and metabolic fitness than after moderate efforts. The mentioned observation proved the positive effects of HIIT on health status in adolescents. Therefore, it is essential to engage youth to participate in physical activity [60]. The HIIT program implemented into physical education lessons increases the general level of physical activity [37]. 

It is necessary to emphasize that the HIIT positive effects occur despite the body composition [39]. However, the dimension of positive changes may depend on some body composition characteristics [25]. Our previous study showed that body composition indices might help establish the optimal value of BMI and FMI to achieve a positive effect in physical fitness improvement after HIIT. Generally, using a ROC curve method effectively describes the cut-off point indicating the phenomenon’s occurrence, e.g., injury risk or failure in a training program based on chosen factors such as physical fitness [28,29] and body composition indices [25,26]. However, there is a lack of observation considering body composition indices concerning blood pressure parameters.

The studies conducted by Ouerghi et al. [61] and Buchan et al. [35] showed positive effects of HIIT in normal and overweight young subjects. In comparison, Mazurek et al. [62] did not provide similar results. Landi et al. [63] show increasing BMI as a hypertension-increasing factor. Chen et al. [64] confirmed this observation that increased BMI is associated with hypertension. Additionally, higher BMI is associated with higher blood pressure in the lean population, but this observation considers mainly women [65]. However, using some body composition indices should use with caution. Especially BMI, which does not reflect the tissue kind; therefore, some conclusions based on it can be overestimated [66]. Thus, the usefulness of other indices is explored. Binthivok et al. [67] showed the efficacy of fat measurements in determining the critical value for increasing the metabolic syndrome risk. Additionally, Wang et al. [68] postulated that measures of adiposity are associated with blood pressure disorders. Korhonen et al. [69] confirmed previous observations where BMI, FMI, and lean mass index (LMI) are informative in blood pressure parameters. Additionally, these authors indicated that increased muscle mass is not beneficial to blood pressure regulation. Zhang & Wang [70] also pointed to BMI and FMI as negative factors for blood pressure values. 

Our study showed that positive changes in blood pressure parameters might be predicted with indices based on muscle mass (SMI and MFR). Therefore, muscle tissue is beneficial to achieving better health results after HIIT. The above study by Korhonen et al. [69] showed a possible negative association between muscle tissue and blood pressure. There is a need to emphasize the difference between our study groups, primarily in age. Muscle mass in maturated groups seems beneficial, especially with lower fat mass. 

We found no previous studies on adolescents that presented the potential of novel body composition indices in predicting the improvement of high-intensity interval training in cardiovascular parameters. Therefore, it is hard to discuss the received cut-off point in our study with other authors. Identifying cut-off point values of the most valuable indices have practical meaning. Therefore, our findings show new directions of examinations and reveal new knowledge in physical activity in physical education lessons. However, due to the lack of similar studies, there is a need to explore this kind of study in wider groups of various ages regarding sex or physical activity. Moreover, there is a need to verify the indicated cut-off values in prospective, longitudinal terms.

We are aware of the limitations of this study. Limited participants from only one school could have affected the results. Several models were very close to significance. The power of the ROC method was circa 46%. Many models received 0.6–0.7 AUC values (*p* = 0.06–0.1). The calculated sample size to receive AUC = 0.73, *p* < 0.05, and 80% power was 57 participants in EG, while 42 females and 31 males took part in the intervention program.

On the other hand, the body composition indices included variables strongly correlated, which could lead to overestimating results. Yet, another shortcoming was the narrow range of adolescents, limited to students of 16 years of age. 

Present study has some strengths. Firstly, the examinations were conducted during typical physical education lesson, during normal schoolwork. Intervention was implemented into PE lesson’s program. Such a design (experimental groups with intervention compared to control groups with typical PE lesson’s program) made it possible to assess the value of the short, time-efficient intensive intervention for adolescents participated in regular school classes. Study sample was homogenous, considering similar subjects, which makes our study reliable to this group. We analyzed separately for males and females when sex is crucial to body composition values. Moreover, the results presented in our study are a novelty. To our knowledge, there is a lack of similar research. 

## 5. Conclusions

The high-intensity interval training intervention is an effective program resulting in positive changes in the cardiovascular system in adolescent males and females. Those who participated in the program were eight times more likely to receive positive changes in blood pressure parameters compared to the control group. The study suggested that body composition indices based on skeletal muscle mass in the formula, SMI and MFR, contributed to positive changes in blood pressure parameters but not BMI and FMI. The higher detection power of the positive changes was related to SBP. The blood pressure indices were poor, except for MAP in females. The males with SMI over 7.84 and MFR over 2.43 and females with SMI over 10.12 and MFR over 1.94 were more likely to receive positive changes in SBP. Intentional modification of the proportions between muscle mass and fat mass through the proper diet may support positive effects on the cardiovascular system after HIIT intervention. The time-efficient interval program implemented in PE lessons improves blood pressure parameters, particularly SBP. However, further studies with greater samples and participant age variations are needed.

## Figures and Tables

**Table 1:** Descriptive statistics and *p*-values derived from *t*-Student tests for differences between EG and CG (in males’ and females’ groups).

	EG				CG			
		Mean	(95% CI)		Mean	(95% CI)		*p*
males	BMI [kg/m^2^]	20.90	19.46	22.33	20.89	19.54	22.23	0.992
	FMI [body fat kg/m^2^]	3.51	2.62	4.41	3.24	2.29	4.20	0.681
	SMI [muscle kg/m^2^]	7.39	6.80	7.97	8.15	7.27	9.03	0.127
	MFR [muscle kg/fat kg]	2.92	2.29	3.56	3.13	2.50	3.75	0.654
	ΔSBP [mm/Hg]	−8.19	−11.41	−4.98	0.48	−1.42	2.37	0.000 *
	ΔDBP [mm/Hg]	−1.71	−4.45	1.03	−1.29	−3.77	1.20	0.825
	ΔPP [mm/Hg]	−6.48	−10.25	−2.72	1.76	−1.63	5.15	0.003 *
	ΔMBP [mm/Hg]	−4.95	−7.27	−2.63	−0.40	−1.82	1.01	0.004 *
	ΔMAP [mm/Hg]	−3.85	−6.16	−1.54	−0.70	−2.36	0.96	0.044 *
females	BMI [kg/m^2^]	20.57	19.97	21.17	21.25	20.12	22.37	0.304
	FMI [body fat kg/m^2^]	5.55	5.11	5.98	6.30	5.54	7.06	0.097
	SMI [muscle kg/m^2^]	9.47	8.78	10.17	9.78	9.10	10.47	0.528
	MFR [muscle kg/fat kg]	1.80	1.62	1.97	1.77	1.54	2.00	0.865
	ΔSBP [mm/Hg]	−4.93	−7.49	−2.36	0.87	−0.57	2.31	0.000 *
	ΔDBP [mm/Hg]	−2.83	−5.68	0.01	0.34	−2.12	2.80	0.091
	ΔPP [mm/Hg]	−2.10	−5.10	0.91	0.53	−1.68	2.74	0.153
	ΔMBP [mm/Hg]	−3.88	−6.14	−1.63	0.61	−1.08	2.29	0.002 *
	ΔMAP [mm/Hg]	−3.53	−5.90	−1.17	0.52	−1.39	2.42	0.008 *

* Statistically significant *p* < 0.05 Abbreviations: body mass index (BMI), fat mass index (FMI), muscle mass index (SMI), mass to fat ratio (MFR), systolic blood pressure (SBP), diastolic blood pressure (DBP), pulse pressure (PP), mid-blood pressure (MBP), and mean arterial pressure (MAP).

**Table 2:** The area under the curve (AUC) and respective cut-off points across all body composition measurements and cardiovascular parameters in experimental (EG) and control groups (CG).

			EG Males				EG Females		
BCI	CVP	AUC	−95% CI	+95% CI	*p*	AUC	−95% CI	+95% CI	*p*
	SBP	0.42	0.21	0.63	0.476	0.47	0.30	0.65	0.767
	DBP	0.59	0.38	0.80	0.392	0.60	0.41	0.79	0.301
BMI	PP	0.46	0.23	0.69	0.712	0.42	0.24	0.59	0.366
	MBP	0.49	0.27	0.71	0.922	0.60	0.40	0.80	0.349
	MAP	0.49	0.29	0.70	0.962	0.55	0.34	0.75	0.642
	SBP	0.45	0.22	0.68	0.648	0.44	0.25	0.63	0.540
	DBP	0.61	0.40	0.81	0.305	0.59	0.39	0.78	0.384
FMI	PP	0.46	0.23	0.68	0.705	0.47	0.29	0.65	0.754
	MBP	0.48	0.25	0.70	0.849	0.57	0.37	0.76	0.488
	MAP	0.51	0.29	0.72	0.963	0.56	0.36	0.76	0.560
	SBP	0.82	0.65	1.00	0.000 *	0.70	0.47	0.92	0.086
	DBP	0.49	0.27	0.71	0.938	0.69	0.49	0.89	0.057
SMI	PP	0.57	0.34	0.80	0.578	0.42	0.24	0.59	0.342
	MBP	0.69	0.47	0.91	0.095	0.59	0.33	0.85	0.482
	MAP	0.60	0.35	0.85	0.425	0.71	0.51	0.91	0.040 *
	SBP	0.70	0.51	0.89	0.039 *	0.72	0.52	0.93	0.035 *
	DBP	0.41	0.20	0.61	0.371	0.62	0.43	0.81	0.220
MFR	PP	0.62	0.39	0.85	0.304	0.44	0.27	0.62	0.516
	MBP	0.64	0.42	0.85	0.218	0.60	0.36	0.83	0.419
	MAP	0.59	0.37	0.80	0.441	0.68	0.48	0.88	0.082
			**CG males**				**CG females**		
	SBP	0.63	0.40	0.87	0.268	0.42	0.26	0.59	0.355
	DBP	0.38	0.13	0.62	0.314	0.51	0.33	0.70	0.903
BMI	PP	0.69	0.46	0.93	0.102	0.42	0.26	0.59	0.348
	MBP	0.60	0.35	0.85	0.435	0.42	0.25	0.59	0.343
	MAP	0.55	0.30	0.81	0.677	0.45	0.28	0.62	0.554
	SBP	0.64	0.40	0.89	0.247	0.43	0.27	0.60	0.442
	DBP	0.51	0.25	0.77	0.943	0.54	0.36	0.71	0.688
FMI	PP	0.68	0.44	0.92	0.150	0.44	0.27	0.60	0.462
	MBP	0.71	0.47	0.94	0.082	0.46	0.29	0.63	0.621
	MAP	0.68	0.44	0.93	0.145	0.50	0.33	0.67	0.983
	SBP	0.57	0.30	0.83	0.616	0.55	0.38	0.73	0.563
	DBP	0.53	0.26	0.79	0.832	0.55	0.39	0.72	0.527
SMI	PP	0.48	0.22	0.74	0.889	0.39	0.23	0.56	0.211
	MBP	0.47	0.21	0.73	0.837	0.62	0.46	0.78	0.137
	MAP	0.47	0.20	0.74	0.843	0.59	0.43	0.76	0.271
	SBP	0.35	0.11	0.58	0.199	0.53	0.36	0.70	0.755
	DBP	0.52	0.26	0.78	0.884	0.51	0.34	0.69	0.881
MFR	PP	0.38	0.13	0.62	0.335	0.51	0.33	0.67	0.999
	MBP	0.31	0.07	0.55	0.118	0.62	0.45	0.78	0.163
	MAP	0.33	0.09	0.57	0.161	0.56	0.40	0.73	0.445

* Statistically significant *p* < 0.05 Abbreviations: Table 1.

**Table 3:** Probabilities of the differences between AUC in comparisons between body composition indices per groups of students.

	SBP	DBP	PP	MBP	MAP
Pair-Wise Comparison			EG Males		
BMI–FMI	0.957	0.574	1.000	0.273	0.481
BMI–SMI	0.041 *	0.342	0.231	0.219	0.431
BMI–MFR	0.134	0.244	0.167	0.189	0.315
FMI–SMI	0.014 *	0.438	0.173	0.044 *	0.217
FMI–MFR	0.134	0.356	0.168	0.084	0.211
SMI–MFR	0.823	0.550	0.486	0.669	0.611
			**EG females**		
BMI–FMI	0.599	0.831	0.427	0.632	0.833
BMI–SMI	0.107	0.545	0.979	0.985	0.315
BMI–MFR	0.139	0.907	0.887	1.000	0.501
FMI–SMI	0.050 *	0.409	0.643	0.873	0.282
FMI–MFR	0.107	0.848	0.860	0.888	0.523
SMI–MFR	0.791	0.455	0.755	0.974	0.729
			**CG males**		
BMI–FMI	0.917	0.274	0.829	0.246	0.180
BMI–SMI	0.287	0.433	0.248	0.524	0.688
BMI–MFR	0.206	0.543	0.178	0.219	0.343
FMI–SMI	0.186	0.886	0.215	0.139	0.212
FMI–MFR	0.203	0.969	0.212	0.082	0.141
SMI–MFR	0.588	0.955	0.526	0.287	0.396
			**CG females**		
BMI–FMI	0.731	0.473	0.657	0.326	0.205
BMI–SMI	0.838	0.755	0.831	0.092	0.243
BMI–MFR	0.509	0.991	0.619	0.208	0.468
FMI–SMI	0.920	0.899	0.732	0.200	0.466
FMI–MFR	0.574	0.896	0.703	0.328	0.686
SMI–MFR	0.293	0.607	0.162	0.941	0.719

* Statistically significant *p* < 0.05. Abbreviations: Table 1.

## Data Availability

Data are available upon request due to ethical restrictions regarding participant privacy. Requests for the data may be sent to the corresponding author.
